# Emotion Regulation Strategies and Persistence of Mood Symptoms in Response to Positive Events: A Follow-Up Study Among Hypomanic Personality Traits, Depressed, and Normal Individuals

**DOI:** 10.1192/j.eurpsy.2025.1080

**Published:** 2025-08-26

**Authors:** E. Ryu, S. Choi

**Affiliations:** Duksung Women’s University, Seoul, Korea, Republic Of

## Abstract

**Introduction:**

Despite the clinical insight that bipolar disorder is associated with specific mood regulation strategies (e.g., positive rumination), this relationship has rarely been confirmed through empirical research. Previous recall-based studies have failed to establish significant links due to potential memory distortion and recall bias. This study addresses these limitations by using a prospective approach to track the direct effects of emotion regulation strategies on mood symptoms in real time and to assess their persistence.

**Objectives:**

The study aimed to: (1) identify differences in emotion regulation strategies in response to positive events among individuals with Hypomanic Personality Traits, depression, and normal controls; and (2) examine how these strategies impact the persistence of mood symptoms over a one-week period.

**Methods:**

90 participants were divided into three groups based on the Hypomanic Personality Scale (HPS) and the Centre for Epidemiological Studies Depression Rating Scale (K-CESD-R): a hypomanic personality trait group (HPS ≥ 29), a depressed group (K-CESD-R > 13), and a healthy control group (HPS ≤ 21, K-CESD-R ≤ 13). Participants completed an initial online survey to assess emotion regulation strategies immediately following positive social and goal-related events. To assess the moderating effect of emotion regulation strategies on mood persistence, participants were asked to complete follow-up surveys at regular intervals over the course of one week to track the sustained emotional impact of the positive event. The nature of each event was pre-classified, and differences in emotion regulation strategies between groups were analyzed. The moderating role of emotion regulation strategies in the relationship between the initial positive mood response and its persistence over time was analyzed using repeated measures analysis of covariance (ANCOVA).

**Results:**

Emotion regulation strategies significantly moderated the relationship between positive events and mood symptoms. The Hypomanic Personality Traits Group predominantly engaged in maladaptive strategies, such as positive rumination, which led to more pronounced positive mood symptoms over time. The Depressed Group used mood dampening strategies, resulting in minimal improvement in mood. Conversely, the Healthy Control Group employed adaptive strategies, such as savoring, which contributed to stable mood.

**Conclusions:**

This study underscores the critical role of emotion regulation strategies in the persistence of mood symptoms. It highlights the negative impact of maladaptive strategies on mood, particularly for individuals with hypomanic traits or depression, suggesting a need for targeted therapeutic interventions. Future research should focus on refining these interventions and exploring their efficacy in diverse populations.

**Disclosure of Interest:**

None Declared

